# Changes in the Top‐Down Control of Planktonic Bacteria in Response to Nutrient Addition and Warming in the Red Sea

**DOI:** 10.1111/1758-2229.70166

**Published:** 2025-08-02

**Authors:** Eman I. Sabbagh, Najwa Al‐Otaibi, Maria Ll. Calleja, Daniele Daffonchio, Xosé Anxelu G. Morán

**Affiliations:** ^1^ Red Sea Research Center (RSRC), Division of Biological and Environmental Sciences and Engineering (BESE) King Abdullah University of Science and Technology (KAUST) Thuwal Saudi Arabia; ^2^ Department of Marine and Coastal Conservation National Center for Wildlife (NCW) Riyadh Saudi Arabia; ^3^ Department of Biology College of Science, Taif University Taif Saudi Arabia; ^4^ Marine Ecology and Systematics Group (MarES), Department of Biology University of Balearic Islands (UIB) Palma de Mallorca Spain; ^5^ Centro Oceanográfico de Gijón/Xixón (IEO, CSIC) Gijón/Xixón Spain

**Keywords:** Autotrophic bacteria, eutrophication, heterotrophic bacteria, heterotrophic nanoflagellates, Red Sea, viruses, warming

## Abstract

Eutrophication and warming impacts on marine bacterioplankton and their top‐down controls (protistan grazers and viruses) are still little known. Here, we evaluated the seasonal variability of the joint impact of nutrient addition and temperature on the abundance of bacterioplankton, heterotrophic nanoflagellates (HNFs) and viruses in Red Sea coastal waters. We conducted four microcosm experiments in which samples were either incubated as such (control, C) or amended with phosphate and nitrate (inorganic, I), glucose (organic, O) or both types of nutrients (mixed, M). Each nutrient treatment was incubated at three temperatures spanning 6°C around ambient values (23°C–33°C). Microbial response ratios (RR, the ratio between the maximum abundance in each nutrient amendment treatment relative to the maximum abundance in the C treatment) were variable, with the most noticeable increases found in the I and M treatments, suggesting an effect mediated by increased primary production. Bacterioplankton showed weak responses to warming, but the RRs of HNFs and viruses in the I treatment tended to increase at higher temperatures. The response of HNFs to the increase in prey was stronger than that of viruses. Our results also suggest that the coupling between heterotrophic bacteria and HNFs will likely increase with future warming.

## Graphical abstract

The responses of planktonic bacteria and their mortality agents (heterotrophic nanoflagellates and viruses) to nutrient additions and temperature were investigated in four seasonal experiments in coastal Red Sea waters. Inorganic and mixed nutrient additions had the greatest effects, with temperature showing a weak impact. However, the trophic link between heterotrophic nanoflagellates and bacteria will likely increase with further regional warming.

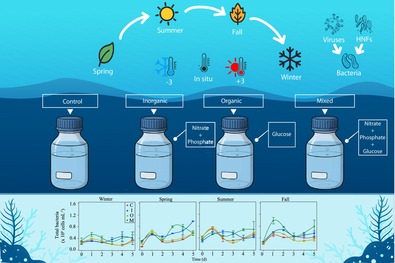

## 1 Introduction

Bacterioplankton represent the major living biomass of the ocean (Whitman et al. 1998) and play key functions in energy and nutrient flow through marine microbial food webs, at both local and global scales (Azam et al. 1983; Carlson et al. 2007). As the rest of the biota, autotrophic and heterotrophic pelagic bacteria have two major modes of regulation: by nutrient availability (bottom‐up control) and by mortality due to protistan grazing and viral lysis (top‐down control) (Billen et al. 1990; Pace and Cole 1994; Tanaka and Rassoulzadegan 2004).

Regarding bottom‐up control, nitrogen (N) and, to a lower extent, phosphorus (P) are the major limiting macronutrients of planktonic communities (Davey et al. 2008; Mills et al. 2008; Moore et al. 2013). Competition for inorganic N and P between phytoplankton and heterotrophic bacteria also takes place (Joint et al. 2002; Gasol et al. 2009), but most of the nutrient requirements of the latter are met through the consumption of labile dissolved organic matter (DOM). Consequently, the availability of labile DOM, commonly measured as carbon (DOC), has frequently been considered the limiting factor for heterotrophic bacterioplankton (Church 2008; Ducklow 1999). *Prochlorococcus* and *Synechococcus* cyanobacteria, and the rest of the components of phytoplankton assemblages, consume inorganic nutrients in a particular proportion that varies between species and environmental conditions (Caron et al. 2000; Church 2008). Nutrient limitation is typically assessed through microbial responses in nutrient enrichment experiments in which the addition of one or combined types of nutrients is compared with controls without addition (e.g., Martínez‐García, Fernández, Calvo‐Díaz, et al. 2010; Teira et al. 2010; Mills et al. 2008). The response of autotrophic and heterotrophic bacteria to nutrient addition can vary due to numerous factors, including changes in the initial community composition and recurrent seasonal changes in the ecosystem's trophic status (Church et al. 2000; Sala et al. 2002; Sánchez et al. 2020).

Collectively known as top‐down control, both grazing by heterotrophic protists (nanoflagellates and to a lower extent ciliates) and viral lysis cause major bacterial losses across aquatic ecosystems (Fuhrman and Noble 1995; del Giorgio et al. 1996; Suttle 2007; Roux et al. 2015). The effects of both mortality factors can directly influence bacterioplankton communities either by size‐selective grazing (Andersson et al. 1986) or host‐specific viral lysis processes (Lønborg et al. 2013) or indirectly through altering organic matter pools (Liu et al. 2015). The effect of protists and viruses can also vary seasonally (e.g., Kaikkonen et al. 2020; Sabbagh et al. 2020) and spatially (e.g., Tsai et al. 2013; Sabbagh et al. 2023), typically following that of their prey or host cells. Several studies have included nutrient manipulation when assessing the role of viral lysis and protistan grazing on microbial food webs with contrasting results. For instance, Williamson and Paul (2004) suggested that inorganic nutrient limitation can enhance viral activities by inducing prophages following the increase in their bacterial host, while other studies have demonstrated that under inorganic nutrient enrichment, the joint effect of grazers and viruses enhanced nutrient regeneration, ultimately increasing bacterial growth (Šimek et al. 2001; Pradeep Ram and Sime‐Ngando 2008).

In addition to trophic interactions, bacterioplankton and their bottom‐up and top‐down controls are also affected by temperature (Vázquez‐Domínguez et al. 2007; Morán et al. 2017). According to future climate scenarios, an increase in sea surface temperature of approximately 1°C–3°C is expected to occur by the end of this century (Richardson et al. 2016; O'Neill et al. 2017), which ultimately will change oceanic carbon fluxes through microbial food webs (Sarmiento et al. 2004). Increasing temperature may result in increased autotrophic (Flombaum et al. 2013) and heterotrophic bacterial abundances (Morán Xosé Anxelu et al. 2015) and alter DOC utilisation (Duarte et al. 2013), potentially resulting in increased bacterial diversity (Ibarbalz et al. 2019), production and respiration (Vázquez‐Domínguez et al. 2007). However, it seems that the effect of increasing temperature is not uniform across ecosystem types or latitude. For example, warming is likely having a greater impact on phytoplankton growth in colder regions (Behrenfeld et al. 2006; Boyce et al. 2010). The current understanding of how the biogeochemistry of tropical ecosystems will change is still limited (Carreira et al. 2024).

While previous studies have tried to understand the impact of bottom‐up (e.g., Joint et al. 2002; Wambeke et al. 2009), top‐down (e.g., Bettarel et al. 2003; Longnecker et al. 2010) or temperature (e.g., Sommer and Lengfellner 2008; Courboulès et al. 2021) controls of bacterioplankton assemblages separately, the joint interplay of the above three factors has rarely been studied. Some examples include the N Mediterranean Sea (Bouvy et al. 2011), the Baltic Sea (Tuomi and Kuuppo 1999) and the S Adriatic Sea (Šolić et al. 2017). In oligotrophic waters, Lewandowska et al. (2014) indicated that the impact of ocean warming on marine plankton and their top‐down control depends on the nutrient conditions. Recent studies in the Red Sea have suggested that the relatively low bacterial abundances found in the coastal waters of its central part are attributed to top‐down control by heterotrophic nanoflagellates (HNFs) and viruses (Ashy and Agustí 2020; Sabbagh et al. 2020, 2023; Silva et al. 2022). To our knowledge, the simultaneous response of bacterioplankton and their top‐down controls to anthropogenic impacts in terms of nutrient loading and warming has never been attempted before in this basin.

To that end, we designed a set of microcosm experiments covering the four seasons (fall 2016 plus winter, spring and summer 2017) with surface water collected from King Abdullah University of Science and Technology (KAUST) Harbor. Microbial plankton samples were either incubated as such (control treatment, C) or amended with phosphate and nitrate (inorganic treatment, I), with glucose (organic treatment, O) or with both types of nutrients (mixed treatment, M). Additionally, each treatment was incubated at three different temperatures spanning 6°C around the temperature recorded in situ. We measured microbial abundances and biomass (autotrophic and heterotrophic bacteria, HNFs and viruses plus chlorophyll *a* as a proxy for phytoplankton) daily for 6 days, focused for further analysis on the response ratios (RRs) (i.e., maximum abundances recorded relative to the C treatment) across the different temperature and nutrient treatments. Based on previous knowledge, we hypothesised that: (i) inorganic nutrient addition would increase phytoplankton biomass (Martínez‐García, Fernández, Álvarez‐Salgado, et al. 2010); (ii) organic nutrient addition would increase the abundance of heterotrophic bacteria (Hitchcock et al. 2010) and consequently those of HNFs and viruses (Bouvy et al. 2011); and (iii) the effect of increased temperature on the stocks and RRs would differ between autotrophic and heterotrophic bacteria and their top‐down controls (Hoppe et al. 2008).

## 2 Material and Methods

### 2.1 Sampling Site

Seawater samples were collected from the Harbour of KAUST (north of Thuwal, Saudi Arabia, 22° 18.412′ N, 39° 6.172′ E), a coastal embayment previously studied in detail regarding the temporal variability of bacterioplankton and their main controlling factors (Silva et al. 2019; Sabbagh et al. 2020, 2023; Ansari et al. 2022). Four microcosm experiments were conducted between 2016 and 2017, aimed at capturing the seasonal variability of the site: fall (started on 19 December 2016), winter (started on 15 February 2017), spring (started on 1 May 2017) and summer (started on 8 August 2017). Surface water (~2 m depth) was collected into four acid‐clean 20 L Nalgene Polycarbonate Clearboy carboys after filtering through a 200 μm sieve, to remove mesozooplankton and transported to the laboratory within 10 min. Temperature and salinity were measured in situ prior to sampling using a portable probe (YSI, Professional Plus) calibrated with a SeaBird 9 CTD probe.

### 2.2 Experimental Design

Samples were immediately carried out to the lab for a series of four treatments: (1) control (C, in situ conditions, no nutrient additions), (2) inorganic (I, 2 μmol L^−1^ of NaNO_3_ and 0.2 μmol L^−1^ NaHPO_4_), (3) organic (O, 5–30 μmol L^−1^ C_6_H_12_O_6_) and (4) mixed (M, a combination of inorganic and organic additions). The additions of nutrients were made based on the maximum annual concentrations known previously in these oligotrophic surface waters, hence aiming at relieving nutrient limitation and stimulating balanced microbial growth. Since we did not observe noticeable responses of microbial abundances to the addition of 5 μmol L^−1^ of glucose in the experiments conducted in fall and winter, we decided to increase its concentration in spring and summer to 30 μmol L^−1^. All treatments (2 L samples) were prepared in duplicates in acid‐washed polycarbonate bottles. Based on the environmental temperature recorded at the time of sampling, each nutrient treatment was incubated at 3 different temperatures (in situ, 3°C above and 3°C below the in situ value) in temperature and light control chambers (Percival, I‐22LLVL) following the natural light: dark regime depending on the sampling period (winter, spring and summer: 12:12 h; fall 11:13 h), with an irradiance of 115 μmol photons m^−2^ s^−1^, tested to be saturating for photosynthesis. Each experiment lasted for 6 days, and sampling was conducted daily at the same local time (10:00 AM) for monitoring changes in inorganic nutrients, DOC, dissolved organic nitrogen (DON) and chlorophyll *a* concentrations, as well as bacterioplankton, viruses and HNFs abundances.

### 2.3 Physico‐Chemical Variables and Chlorophyll *a*

Subsamples for inorganic nutrients were collected in 15 mL Falcon tubes after filtering through 0.7 μm nominal pore‐size glass fibres filter (Whatman GF/F) that had been pre‐combusted at 470°C for 5 h. Samples were then analysed using a Seal Analytical segmented flow analyser (Analytical Core Lab, KAUST) to measure the concentration of nitrate (NO_3_^−^), nitrite (NO_2_^−^) and orthophosphate, hereinafter phosphate (PO_4_^3−^) following the procedure of Hansen and Koroleff (1999).

Subsamples for DOC and total dissolved nitrogen (TDN) were collected in amber glass tubes after filtering through pre‐combusted Whatman GF/F filters were then acidified with 200 μL of phosphoric acid (85%) to a pH 1–2 and kept at 4°C until analysis. A high temperature catalytic oxidation (HTOC) on a Shimadzu TOC‐L was used to analyse the samples following the procedures previously described in Calleja et al. (2018). DON was calculated as DON = TDN − DIN (DIN = nitrate + nitrite).

Chlorophyll *a* concentrations were obtained by filtering 200 mL of seawater sequentially through three polycarbonate filters (Millipore, 47 mm diameter) of decreasing pore size: 20, 2 and 0.2 μm. Filters were stored at −20°C until further analysis. Chlorophyll *a* pigment was extracted by sonicating the filters in 90% acetone and incubating at +4°C for 24 h. A Trilogy fluorometer (Turner design) calibrated with a chlorophyll *a* standard ( *Anacystis nidulans* , Sigma‐Aldrich) was used to measure chlorophyll *a* fluorescence. The total concentration of chlorophyll *a* was calculated as the sum of the three size classes.

### 2.4 Microbial Abundances

A FACSCanto II (BD Bioscience) flow cytometer was used for all flow cytometric analyses following the methods described in Gasol and Morán (2015). Latex fluorescence beads (1 μm diameter, Molecular Probes ref. 17154‐10) were used as an internal standard for the various light scatter and fluorescence signals. The flow rate (μL min^−1^) was estimated by running 1 mL of Milli‐Q water with 10 μL of the working beads solution for 5 min and weighing it before and after that time. FCS Express 5 (De Novo Software) was used for all post‐acquisition analyses.

For autotrophic and heterotrophic bacteria, 1800 μL of sample were fixed with 1% paraformaldehyde +0.05% glutaraldehyde (final concentration), incubated in the dark for 10 min and stored at −80°C until further analysis. Autotrophic bacterial abundances were analysed in 600 μL of thawed samples after adding 10 μL of the beads solution and run in the flow cytometer at high flow rate. *Prochlorococcus* and two groups of *Synechococcus* of differing orange fluorescence (low and high, LF‐*Synechococcus* and HF‐*Synechococcus*, respectively) were distinguished based on their phycoerythrin (orange fluorescence) and chlorophyll (red fluorescence) pigments content and cell size (estimated from the side scatter or SSC signal) as in Sabbagh et al. (2020). Following the procedures of Gasol and Morán (2015), heterotrophic bacterial abundances were analysed in 400 μL of thawed samples previously stained with 4 μL of SYBR Green I (Molecular Probes), diluted in 10× dimethylsulfoxide (DMSO) and incubated in the dark for 5 min. Heterotrophic bacteria samples were run at low flow rate after the addition of 10 μL of the beads solution. Two groups of heterotrophic bacteria, low nucleic acid bacteria (LNA) and high nucleic acid bacteria (HNA), were distinguished based on their green fluorescence (indicative of nucleic acid content) and SSC signals. Total heterotrophic bacterial abundance was calculated as the sum of LNA + HNA cells.

For heterotrophic nanoflagellates (HNFs), 4 mL of sample was fixed with 0.7% glutaraldehyde (final concentration), incubated in the dark for 10 min and stored at −80°C. 1000 µL of the thawed samples was stained with 10 μL of SYBR Green I (Molecular Probes, diluted 10× in DMSO), incubated in the dark for 5 min and run in the flow cytometer after the addition of 10 μL of beads solution. HNFs were identified in red and green fluorescence versus SSC plots, following the protocol of Christaki et al. (2011).

For viruses, used here to encompass all virus‐like particles distinguished by flow cytometry, we followed the protocol optimised by Brussaard (2004). Hence, 1500 μL of seawater were fixed with 4% glutaraldehyde (final concentration), incubated in the dark for 10 min and stored at −80°C for further analysis. 25 µL microlitres of the thawed samples were diluted in 475 μL 1× Tris‐EDTA (TE) buffer, stained with 5 μL of SYBR Green I (Molecular Probes, diluted 100× in MilliQ water), incubated at 80°C for 10 min, cooled down in the dark for 10 min and run in the flow cytometer after the addition of 10 μL of beads solution. According to the green fluorescence and SSC signals, we distinguished three subgroups of viruses characterised by low (V1), medium (V2) and high (V3) nucleic acid content (Brussaard 2004; Sabbagh et al. 2020). A control was run daily before the analysis by adding 5 μL of SYBR Green I to 500 μL of 1× TE buffer. Total viruses were calculated as the sum of V1, V2 and V3 group abundances.

### 2.5 Statistical Analyses

Using just duplicates per nutrient and temperature treatment rather than more replicates is a potential limitation. However, the observed variability was rather low, with microbial abundances mean coefficients of variation (seasonal CVs were first calculated for each group considering all temperature and nutrient values over time) consistently below 18%, ranging from 12.2% ± 3.1% (*Synechococcus*) to 17.6% ± 9.5% (viruses). The RR of the different microbial populations to the three nutrient addition treatments was calculated as the ratio between the maximum abundances reached in the I, O and M treatments divided by the maximum abundance reached in the C treatment. Maximum abundances were typically reached 1–2 days earlier in the warmest temperatures compared with the coolest ones. The use of RRs is an appropriate way of circumventing differences in microbial community composition (Ansari et al. 2022), since the effect of nutrient additions is considered relative to each seasonal control value, regardless of the initial conditions. Normality was met (Shapiro–Wilk *W* test, *p* > 0.05) in 63% of the seasonal distributions of RRs (10 out of 16) and homoscedasticity in all of them (*Synechococcus*, *p* = 0.208; heterotrophic bacteria, *p* = 0.436; HNFs, *p* = 0.062; Viruses, *p* = 0.051). We used *t*‐tests to detect significantly positive or negative RR values against 1.0 (i.e., no change of a given nutrient amendment treatment relative to the control). Other statistical analyses included paired *t*‐tests, Pearson's correlations, analysis of variance (ANOVA) and covariance (ANCOVA) and post hoc Tukey HSD tests. All figures and statistical analyses were performed with JMP (Pro 15) software.

## 3 Results

### 3.1 Physico‐Chemical In Situ Conditions

The physico‐chemical conditions at the onset of the experiments are presented in Table 1. Temperature showed the expected seasonality with lowest and highest values in winter and summer, respectively, while salinity showed a similar but less marked seasonal pattern. Nitrate concentration was lower in winter and spring than in summer and fall, where it exceeded 6 μmol L^−1^ (Table 1). Phosphate concentrations were well below 0.1 μmol L^−1^ in all seasons (Table 1). The ratio of inorganic nitrogen (nitrate + nitrite) to phosphorus (N:P) ranged from 40 in winter to 266 in summer. The maximum DOC concentration was found in summer (103.09 μmol C L^−1^), with similar values in spring and fall and minimum concentration in winter (77.37 μmol C L^−1^, Table 1). DON concentration followed a different pattern, with maximum values in winter and relatively lower values through the rest of the year (Table 1). Total chlorophyll *a* concentrations showed little variability, ranging from 0.38 μg L^−1^ in spring to 0.43 μg L^−1^ in winter (Table 1). Based on fractionated chlorophyll *a* measurements, the picophytoplankton size‐class was dominant in all seasons, with a mean value of 46% ± 12%, followed by nanophytoplankton (35% ± 9%) and microphytoplankton (20% ± 13%).

**TABLE 1:** In situ values of selected environmental variables in the four seasonal experiments.

	Winter	Spring	Summer	Fall
Temperature (°C)	22.9	30.1	33.4	26.0
Salinity	38.0	39.3	39.8	38.2
Nitrate (μmol L^−1^)	1.54	2.59	6.54	6.11
Phosphate (μmol L^−1^)	0.05	0.05	0.03	0.06
DOC (μmol C L^−1^)	77.37	86.06	103.09	82.07
DON (μmol N L^−1^)	9.6	8.1	8.0	8.4
Total chlorophyll *a* (μg L^−1^)	0.43	0.38	0.29	0.31

*Note:* Mean ± SD of duplicate samples.

### 3.2 Initial Microbial Abundances

**FIGURE 1:**
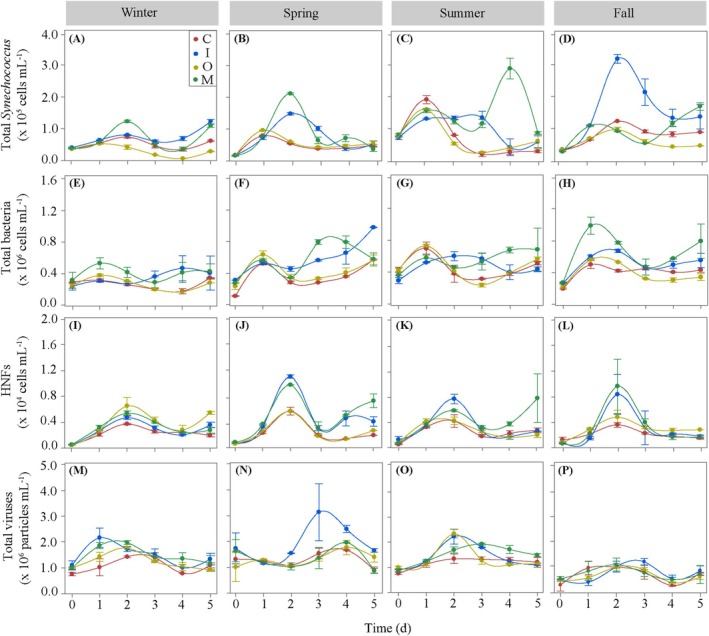
Dynamics of total *Synechococcus* (A‐D), total heterotrophic bacteria (E–H), heterotrophic nanoflagellates (HNFs) (I–L) and viruses (M–P) at in situ temperature in the different nutrient treatments. (C) control; (I) inorganic; (O) organic; (M) mixed. Error bars represent SD of duplicate samples.

Picophytoplankton were numerically dominated by *Synechococcus* cyanobacteria in all seasons, with *Prochlorococcus* only present in winter in low numbers (2.11 × 10^4^ cells mL^−1^). The initial total abundance of *Synechococcus* (LF plus HF groups) ranged from 1.48 to 8.17 × 10^4^ cells mL^−1^ and was significantly higher in summer (ANOVA, *p* < 0.0001, Tukey HSD test, Figure 1A–D). LF *Synechococcus* were found year‐round, with a mean contribution of 70% ± 30% to total *Synechococcus* counts, while HF *Synechococcus* dominated in fall (71% ± 1%) and were completely absent in summer (Figure S1A–D). In winter, spring and fall, the relative abundance of LF *Synechococcus* tended to decrease as the incubation progressed. The total abundance of heterotrophic bacteria ranged from 1.28 to 5.78 × 10^5^ cells mL^−1^, with minimum numbers in spring and maximum in summer (ANOVA, *p* = 0.0007, Tukey HSD test, Figure 1E–H). The heterotrophic bacterioplankton community was dominated by HNA cells (mean initial contribution of 63% ± 7%) in all seasons except in fall, where LNA contributed 55% to total heterotrophic bacterial counts (Figure S1E–H). HNFs initial abundances ranged from 0.48 to 1.73 × 10^3^ cells mL^−1^ and did not show any significant differences between seasons (ANOVA, *p* > 0.05, Figure 1I–L). The total abundances of viruses ranged from 0.20 × 10^7^ in fall to 1.79 × 10^7^ viruses mL^−1^ in spring (ANOVA, *p* = 0.03, Tukey HSD test, Figure 1M–P). Viruses were dominated by the V1 group in spring and fall (50% and 52%, respectively) and by the V2 group in winter and summer (48% and 53%, respectively), while the V3 group contributed little to total viral counts, from (7% in spring to 174% in fall, Figure S1I–L).

### 3.3 Effect of Nutrient Addition on Microbial Abundances at In Situ Conditions

The dynamics of microbial groups abundances at in situ temperature in each nutrient treatment and season are presented in Figure 1, while Figure S1 shows the relative contribution of specific groups, namely LF *Synechococcus*, HNA bacteria and V1 viruses. Compared with the control treatment, the total abundances of *Synechococcus* reached significantly higher maximum values in both the I and M treatments in winter, spring and fall (ANOVA, *p =* 0.005, *p =* 0.0001, *p =* 0.0002, respectively, Tukey HSD tests) and the M treatment in the same seasons (ANOVA, winter: *p =* 0.005, spring: *p* < 0.0001, fall: *p* = 0.010, Tukey HSD tests). No differences between treatments were found in the contribution of LF *Synechococcus* in any season (ANOVA, *p* > 0.05, Figure S1A–D). Total heterotrophic bacteria abundance showed a consistent peak after just 1 day in all treatments and seasons and then followed different dynamics (Figure 1E–H). Significantly higher maximum heterotrophic bacterial abundances were reached in the I treatment in spring (ANOVA, *p =* 0.01, Tukey HSD test, Figure 1F) and the M treatment in fall (ANOVA, *p* = 0.01, Tukey HSD test, Figure 1H) compared to the control. A significantly higher HNA cells contribution was found only in the M treatment in fall (ANOVA, *p* = 0.001, Tukey HSD test, Figure S1H). Although HNF abundances consistently peaked in all treatments at Day 2 (Figure 1I–L), significantly higher than the control maximum abundances were found in the I and M treatments in spring only (ANOVA, *p* = 0.001 and *p* = 0.003, respectively, Tukey HSD tests, Figure 1J). The dynamics of total viral abundance varied between seasons, with their maximum absolute and relative (i.e., the contribution of V1, V2 and V3 subgroups) abundances not showing any significant difference compared to the C treatment (ANOVA, *p* > 0.05, Figures 1M–P and S1I–L).

**FIGURE 2:**
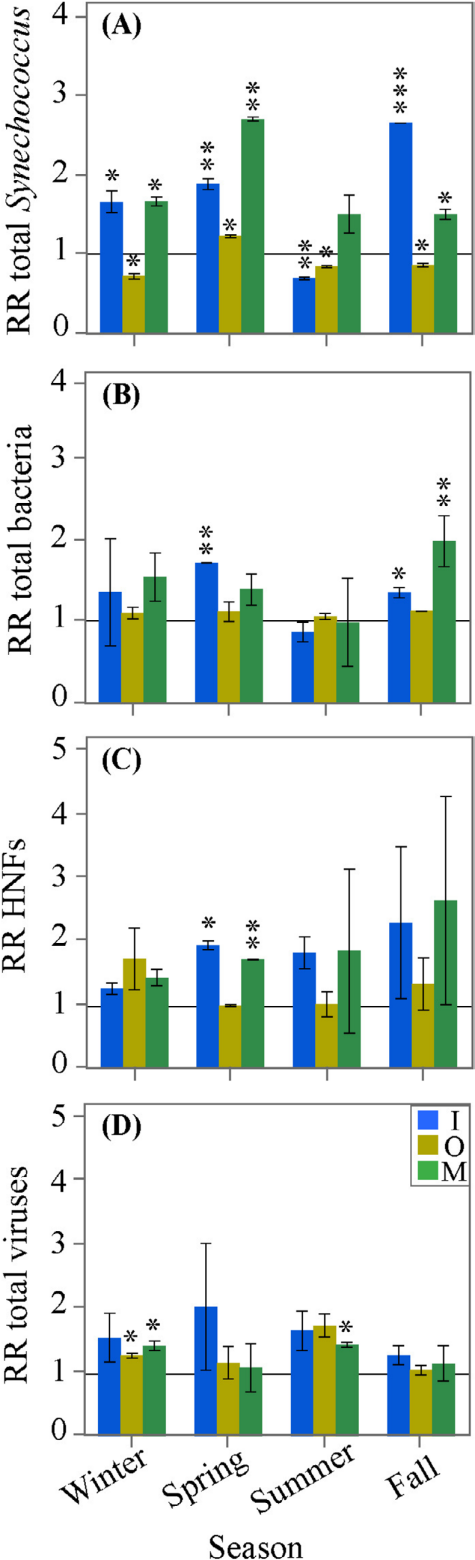
Mean seasonal values of the response ratio (RR) of (A) total *Synechococcus*, (B) total heterotrophic bacteria, (C) heterotrophic nanoflagellates (HNFs) and (D) total viruses at in situ temperature in the different nutrient addition treatments (codes as in Figure 1). Error bars represent the SD of duplicate samples. The horizontal line represents a RR of 1.0 (no change relative to the C treatment). Asterisks indicate a RR significantly different from 1 (*t*‐test: *p < 0.05; **p < 0.01; ***p < 0.001).

For a better assessment of the effect of nutrient additions on bacterioplankton and their top‐down controls, we focused on the RRs. Total *Synechococcus* and HNFs were the groups showing the highest RRs, exceeding twice the value of 2 (i.e., double the control value). Total *Synechococcus* RRs were significantly positive in the I and M treatments in all seasons but summer, but were significantly negative in the O treatment except in spring (Figure 2A). The RRs were inconsistent among treatments and seasons for the two groups of *Synechococcus* (Figure S2A,B). The RRs of total heterotrophic bacteria were significantly > 1 only in the I treatment in spring and fall and in the M treatment in summer (Figure 2B). The RRs of HNA heterotrophic bacteria were typically higher than those of LNA cells (paired *t*‐test, *p* = 0.001, *n* = 72), but the two groups responded differently to the different treatments. LNA RRs were significantly higher than 1 in the I treatment in spring and in the M treatment in fall, and lower in the O treatment in winter and in the M treatment in summer (Figure S2C). On the contrary, the RRs of HNA presented the highest positive value in the O treatment in summer and fall, followed by the I and M treatments in both spring and fall, but were negative in the I treatment in summer (Figure S2D). Although all RRs of HNFs were positive in the I and M treatments, significant responses were only found in spring (Figure 2C). Total viruses showed significantly higher RRs in the O treatment in winter and in the M treatment in both winter and summer (Figure 2D). Although no clear pattern was shown by the different subgroups, the V1 subgroup showed a RR significantly higher than 1 in the O treatment in winter (Figure S2E), while V2 was significantly higher than 1 in the I treatment in spring (Figure S2F), and the V3 subgroup in the I treatment in both summer and fall, and the M treatment only in summer (Figure S2G).

### 3.4 Effect of Temperature on Microbial Responses

Experimental warming in the C treatment did not result in consistent responses across all groups and seasons, but the increase in maximum abundance with temperature (cell/viruses °C^−1^) tended to be higher in spring and summer than in winter and fall, where the maximum abundance of heterotrophic groups even showed decreases with rising temperatures (Table 2). Interestingly, the increase in *Synechococcus* maximum abundance with warming was higher with higher initial nitrate concentrations (Table 2) and was significantly correlated with the nitrate: phosphate ratio (*r* = 0.99, *p* = 0.011, *n* = 4), while the response of heterotrophic bacteria was also significantly and positively correlated with initial DOC concentration (*r* = 0.99, *p* = 0.011, *n* = 4). When all nutrient treatments were considered, most of the temperature responses of *Synechococcus*, heterotrophic bacteria, HNFs, and viruses maximum abundances were positive (88%, Table 2), although only a few of them were significant given the low number of samples used for the calculations (*n* = 3/6). However, with all data pooled, the maximum abundance changes with temperature of heterotrophic bacteria covaried significantly with those of HNFs (*r* = 0.75, *p* = 0.0008, *n* = 16) and *Synechococcus* (*r* = 0.73, *p* = 0.0014), but not with those of viruses (*p* = 0.60).

**TABLE 2:** Temperature dependence (i.e., slopes of the ordinary least squares linear regressions, cell/virus numbers °C^−1^) of the maximum abundances of *Synechococcus*, heterotrophic bacteria, heterotrophic nanoflagellates (HNFs) and viruses in each season and nutrient treatment.

Season	Treatment	*Synechococcus*	Heterotrophic bacteria	HNFs	Viruses
Winter	Control	**3.62 × 10** ^**3**^	−1.03 × 10^4^	3.00 × 10^0^	−2.49 × 10^5^
	Inorganic	**1.78 × 10** ^**4**^	**3.75 × 10** ^**4**^	**−9.50 × 10** ^**1**^	**1.76 × 10** ^**6**^
	Organic	**2.60 × 10** ^**3**^	1.99 × 10^2^	−1.31 × 10^2^	**3.17 × 10** ^**6**^
	Mixed	**1.71 × 10** ^**4**^	**3.59 × 10** ^**4**^	1.85 × 10^2^	**3.69 × 10** ^**5**^
Spring	Control	**4.46 × 10** ^**3**^	**1.88 × 10** ^**4**^	**7.65 × 10** ^**2**^	4.47 × 10^4^
	Inorganic	**−6.95 × 10** ^**3**^	**1.07 × 10** ^**4**^	**7.06 × 10** ^**2**^	6.36 × 10^5^
	Organic	**7.67 × 10** ^**3**^	8.39 × 10^3^	−1.65 × 10^2^	5.12 × 10^5^
	Mixed	**−9.21 × 10** ^**3**^	**6.22 × 10** ^**4**^	1.69 × 10^2^	2.19 × 10^5^
Summer	Control	**1.32 × 10** ^**4**^	**8.11 × 10** ^**4**^	**6.20 × 10** ^**2**^	**9.47 × 10** ^**5**^
	Inorganic	**5.73 × 10** ^**4**^	**1.37 × 10** ^**5**^	**1.58 × 10** ^**3**^	**2.91 × 10** ^**6**^
	Organic	**2.01 × 10** ^**4**^	**7.77 × 10** ^**4**^	**3.12 × 10** ^**2**^	6.55 × 10^5^
	Mixed	**6.83 × 10** ^**4**^	**1.12 × 10** ^**5**^	**1.25 × 10** ^**3**^	**3.78 × 10** ^**6**^
Fall	Control	6.31 × 10^3^	−6.32 × 10^3^	**−1.21 × 10** ^**2**^	**9.84 × 10** ^**5**^
	Inorganic	**1.36 × 10** ^**3**^	**−5.35 × 10** ^**4**^	6.50 × 10^1^	8.98 × 10^4^
	Organic	−1.14 × 10^2^	7.03 × 10^2^	2.50 × 10^1^	1.72 × 10^5^
	Mixed	**2.40 × 10** ^**4**^	6.00 × 10^3^	1.89 × 10^2^	**9.87 × 10** ^**6**^

*Note:* Highlighted in bold are those regressions with a coefficient of determination (*r* ^2^) higher than 0.5.

**FIGURE 3:**
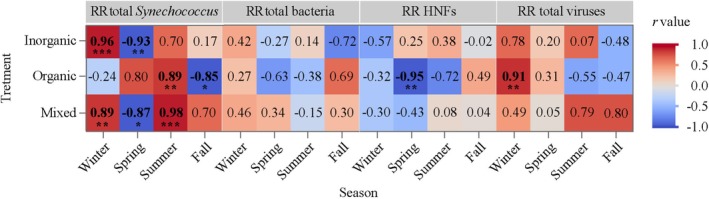
Heatmap showing the Pearson correlation coefficients between the RRs of the different microbial groups and temperature for each nutrient treatment and season. Asterisks indicate significant correlation (*p < 0.05; **p < 0.01; ***p < 0.001).

The effect of temperature on the RRs of the various microbial groups was initially investigated by calculating Pearson's correlations for each seasonal experiment using the respective temperature ranges rather than the broad categories of −3°C, in situ temperature and +3°C of the 3‐way ANOVA described later. Temperature ranges were 19.9°C–25.9°C in winter, 27.1°C–33.1°C in spring, 30.4°C–36.4°C in summer, and 23.0°C–29.0°C in fall. In general, few consistent responses were observed (Figure 3). For instance, the RRs of *Synechococcus* correlated positively with temperature in both the I and M treatments in winter but negatively in spring, while the positive correlations in summer were only significant in the M treatment. Significant correlations in the O treatment were positive in summer and negative in fall (Figure 3). Although we found both positive and negative correlations of total heterotrophic bacterial RRs with temperature, none of them were significant (*p* > 0.05, Figure 3). In the case of HNFs, the correlation between the RR and temperature was negative and significant only in the O treatment in spring (Figure 3). The response of total viruses RRs to temperature was also inconsistent, and significantly positive only in the O treatment in winter (*p* = 0.01, Figure 3).

**FIGURE 4:**
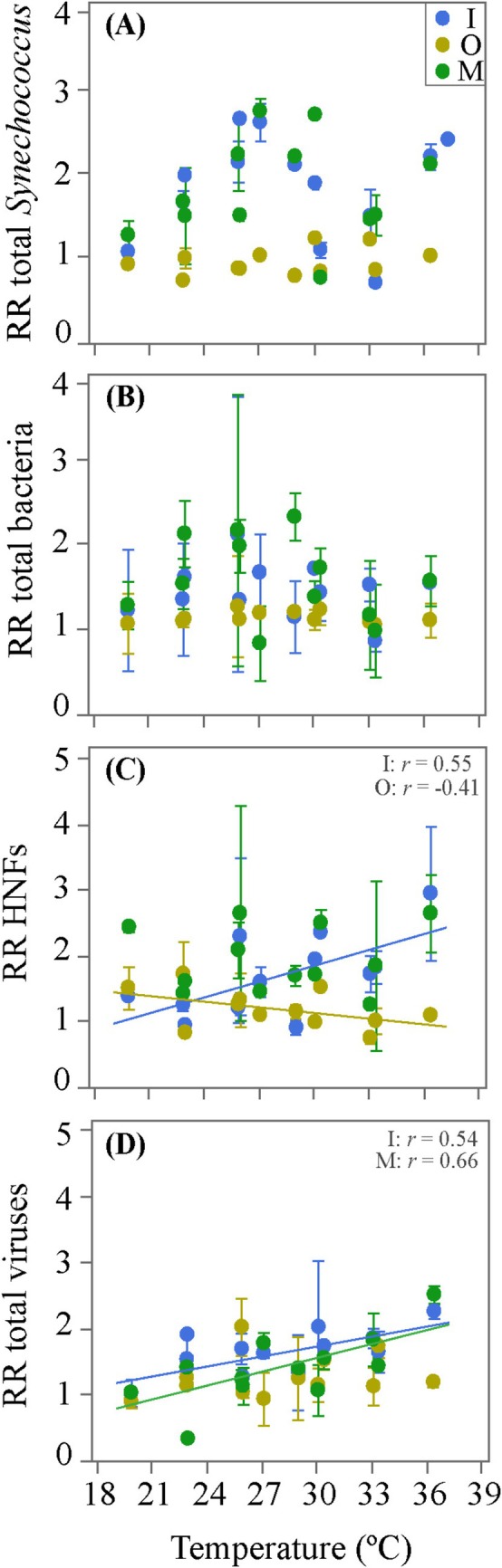
Relationships between the RRs of the different microbial groups and temperature pooling data for each nutrient amendment treatment. (A) total *Synechococcus*, (B) total heterotrophic bacteria, (C) heterotrophic nanoflagellates (HNFs) and (D) total viruses.

In order to assess the existence of general relationships between the RRs of the different microbial groups and temperature, we pooled all seasonal and incubation temperature data for each nutrient amendment treatment, therefore resulting in a temperature range of more than 16°C (19.9°C–36.4°C). Neither the RRs of total *Synechococcus* or heterotrophic bacteria abundances did show any significant relationship with temperature for any of the 3 nutrient additions (Figure 4A,B). Interestingly, the RRs of HNF showed a positive response to warming in the I treatment (*r* = 0.55, *p* = 0.005, *n* = 24, Figure 4C) and a negative one in the O treatment (*r* = −0.41, *p* = 0.04, *n* = 24, Figure 4C). The RRs of total viruses increased significantly with increasing temperature in both the I and M treatments (*r* = 0.54, *n* = 23, *p* = 0.006 and *r* = 0.66, *p* = 0.007, *n* = 22, respectively, Figure 4D).

**FIGURE 5:**
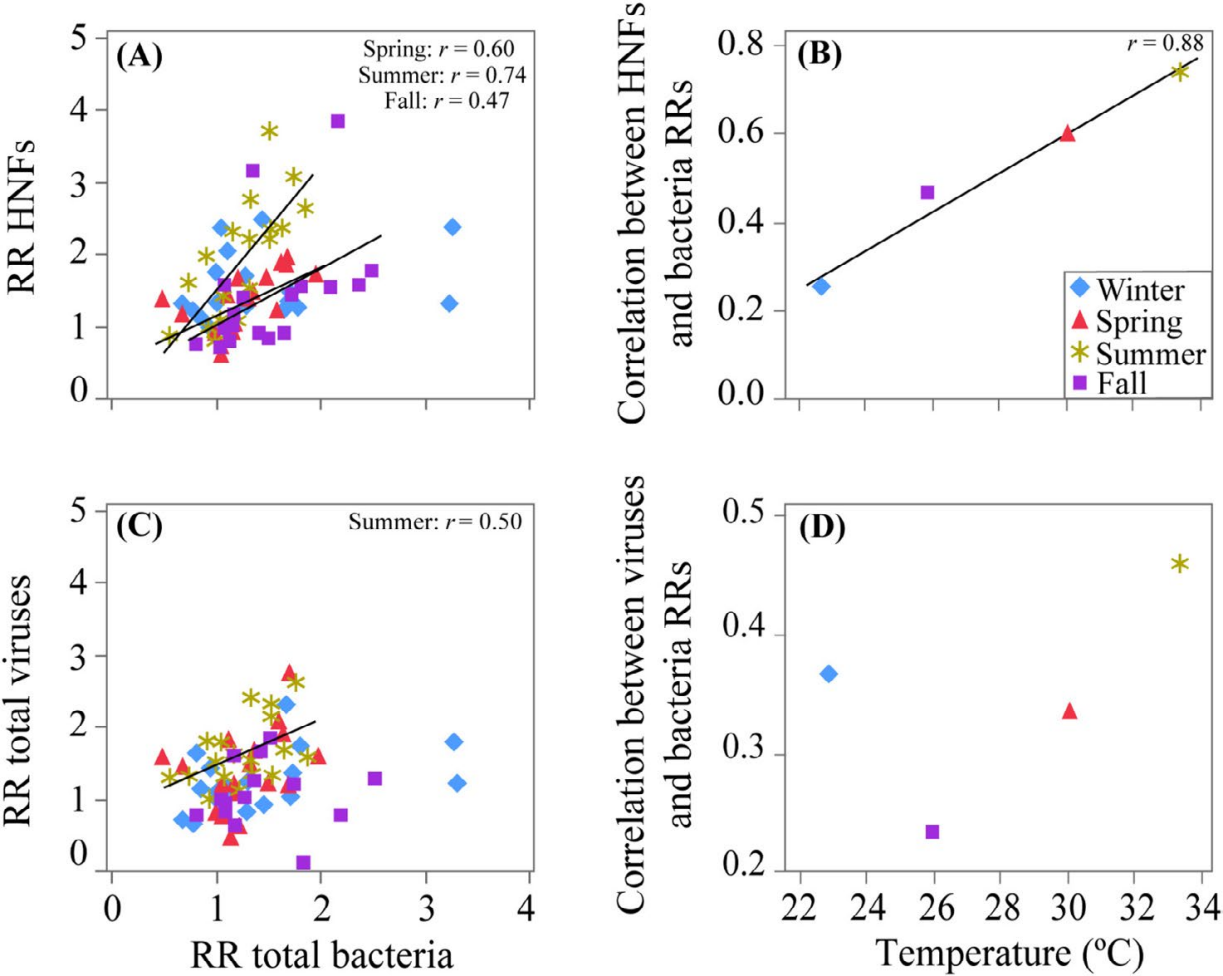
Relationship between the RRs of total heterotrophic bacteria and the RRs of (A) HNFs and (C) total viruses, for each season (pooling all nutrient treatments and temperature data). The relationship between the seasonal correlation coefficients obtained from plot A and plot C and the in situ temperature of (B) the RRs of HNF and heterotrophic bacteria, and (D) the RRs of total viruses and heterotrophic bacteria.

We additionally explored the possible coupling between the responses of heterotrophic bacteria and their top‐down controls by pooling data for each seasonal experiment (Figure 5A,C). No significant relationships were found for cyanobacteria, either for *Prochlorococcus* in winter or for total *Synechococcus* or any of the two groups in any season. However, the RRs of HNFs showed a positive correlation with those of heterotrophic bacteria in spring, summer and fall (Figure 5A). The slopes of the linear regression were significantly different (ANCOVA, *p* < 0.05), with a significantly higher value in summer (1.66) compared to spring and fall (0.64 and 0.75, respectively). The RRs of total viruses were also positively correlated with the RRs of heterotrophic bacteria, but the correlation was significant only in summer (Figure 5C). Figure 5B,D show the seasonal correlation coefficients (all *r* values regardless of their significance) obtained from Figure 5A,C versus the temperature recorded in situ for each season. In spite of having only 4 values, we found a very strong and positive relationship of the degree of coupling between the RRs of heterotrophic bacteria and HNFs (i.e., the correlation coefficients taken from Figure 5A including the non‐significant winter value) with temperature (*r* = 0.99, *p* = 0.01, *n* = 4, Figure 5B). The relationship of the correlation between the RRs of heterotrophic bacteria and viruses (Figure 5C) with temperature was not significant although it was markedly positive between 26°C and 34°C (Figure 5D).

Finally, 3‐way ANOVAs of the RRs of the four microbial groups and total chlorophyll *a* as a surrogate for phytoplankton biomass with season, nutrient and temperature as factors showed significant, although different, effects (Table 3). Thus, the RRs of *Synechococcus*, HNFs, and total chlorophyll *a* were significantly higher in spring, summer and fall, respectively. Nutrient effects previously presented were also apparent for all microbial groups except viruses, with significantly higher RRs in the M and/or the I than the O treatments. Using the broad categories of −3°C, in situ value, and +3°C, temperature was a significant factor only for phytoplankton, both for total chlorophyll *a* and *Synechococcus* cyanobacteria. The temperature dependence of absolute cell counts change and RRs was captured in more detail by the analyses previously shown (Table 2 and Figures 3, 4, 5).

**TABLE 3:** Results of the 3‐way ANOVA to test the significance of variations in microbial RRs associated with sampling season, the various nutrient amendments, and temperature treatments.

Source	df	Heterotrophic bacteria		*Synechococcus*		HNF		Viruses	Chlorophyll *a*	
Seas(on)^a^	3	1.28		46.48***	2 > 4 > 1 > 3	5.08**	3 > 4 = 2	1.16	128.59***	4 > 2 = 3 > 1
Nutr(ient)^b^	2	5.32**	3 > 2	231.61***	3 = 1 > 2	15.42***	3 = 1 > 2	1.66	229.69***	1 = 3 > 2
Temp(erature)^c^	2	1.29		15.29***	3 > 2 = 1	0.30		1.06	21.70***	2 = 3 > 1
Seas × nutr	6	1.96		13.02***		3.01*		0.42	36.04***	
Seas × temp	6	1.28		34.10***		3.84**		2.66*	36.67***	
Nutr × temp	4	0.26		4.10**		0.38		0.19	3.52*	
Seas × nutr × temp	12	0.32		15.44***		0.84		0.49	12.40***	

*Note:* *F* ratios are shown with an indication of significance: *p < 0.05, **p < 0.01, ***p < 0.001, and Tukey HSD post hoc comparisons of single sources to the right of each variable. Heterotrophic bacteria, total heterotrophic bacteria abundance; *Synechococcus*, total *Synechococcus* abundance; HNF, heterotrophic nanoflagellates abundance; viruses, total viruses abundance; Chlorophyll *a*, total chlorophyll *a* concentration.

^a^ 1, winter; 2, spring; 3, summer; 4, fall.

^b^ 1, inorganic (I); 2, organic (O); 3, inorganic + organic (M).

^c^ 1, −3°C; 2, in situ; 3, +3°C.

## 4 Discussion

This study contributes to the limited body of studies investigating the joint effect of eutrophication and warming on bacterioplankton and their top‐down controls. Such investigations are lacking for many regions, but especially in tropical waters. To date, the above factors have been mostly studied separately (e.g., Øvreås et al. 2003; Berninger and Wickham 2005; Sarmento et al. 2010; Tsai et al. 2015). The most noticeable responses of microbial plankton to nutrient additions were found in the I and M treatments, significantly higher than in the O treatment both for autotrophs and heterotrophs, except viruses (Figure 2 and Table 3), suggesting that the effect was a sequential enhancement of trophic interactions starting with increased primary production. Experimental warming also impacted the various microbial groups, but it did so in a less consistent way (cf. for instance Tables 2 and 3 and Figure 3). Although our data suggest that nutrient availability was a more important factor than temperature in regulating the stocks of tropical bacterioplankton and their top‐down controls, we found that the coupling between the responses of heterotrophic bacteria and HNFs to nutrient additions became tighter at warm ambient temperatures (Figure 5B).

The initial physico‐chemical conditions at the study site reflected the seasonality of these shallow waters (Silva et al. 2019; Sabbagh et al. 2020, 2023). The persistent limitation of planktonic assemblages by phosphorus at KAUST Harbor (Silva et al. 2019; Sabbagh et al. 2020) was also found in our experiments, with in situ N:P ratios well above 16 (from 40 to 266). Although variable (Table 1), mean DOC concentrations (87.1 ± 10.4 μmol C L^−1^) aligned well with previously reported values (Silva et al. 2019; Ansari et al. 2022; Sabbagh et al. 2023). Total chlorophyll *a* showed similarly low concentrations (Silva et al. 2019; Sabbagh et al. 2020; Ansari et al. 2022), with just winter values exceeding 0.4 μg L,^−1^ and phytoplankton was dominated by the smallest size‐class (< 2 μm), which contributed on average 45% ± 12% to total concentration, similarly to previous reports (Brewin et al. 2019; Sabbagh et al. 2020). The very low initial abundance of *Prochlorococcus* in winter (< 2 × 10^4^ cells mL^−1^) was consistent with higher resolution (weekly and monthly) samplings at the site (Sabbagh et al. 2020, 2023; Ansari et al. 2022). Also, similar to previous studies, heterotrophic bacterial initial abundances were well below 10^6^ cells mL^−1^ (Silva et al. 2019; Al‐Otaibi et al. 2020; Sabbagh et al. 2020, 2023; Ansari et al. 2022), lower than the values found elsewhere (e.g., Li 1998; Teira et al. 2019; Gómez Letona 2023). Finally, both HNFs and total viruses initial abundances in the 4 experiments matched those previously found in the same waters (Sabbagh et al. 2020, 2023).

Bottom‐up control is a key factor regulating bacterioplankton abundances and dynamics in aquatic ecosystems (Ducklow 1999; Gasol et al. 2002; Fuhrman et al. 2015). Microcosm and mesocosm manipulation experiments have put the effort to understand the ecological interactions between phytoplankton and heterotrophic bacteria and nutrient availability in different oceanic regions (e.g., Martínez‐García, Fernández, Calvo‐Díaz, et al. 2010; Teira et al. 2010; Barcelos e Ramos et al. 2017), but they have been seldom conducted in the Red Sea (Berninger and Wickham 2005; Pearman et al. 2017; Coello‐Camba et al. 2020), including bacterioplankton mortality agents. Since we did not observe any noticeable response of microbial abundances to the addition of 5 μmol L^−1^ of glucose in the O and M treatments in the first two experiments conducted in fall and winter, we decided to increase its concentration in spring and summer to 30 μmol L^−1^. The addition of 25 μmol L^−1^ of glucose in spring and summer on top of the 5 μmol L^−1^ used in the first two experiments did not change significantly heterotrophic bacterial responses compared to those found in winter and fall, and the RRs were relatively comparable in all seasons (Figure 2B). Indeed, RR values were significantly lower in the O than in the I and M treatments for heterotrophic bacteria and the rest of the microbial groups (Table 3). This lack of response to the sole addition of glucose as a labile DOC compound was somewhat unexpected (Thingstad et al. 1997), since most studies similar to ours have found a significant effects (e.g., Martínez‐García, Fernández, Calvo‐Díaz, et al. 2010; Martínez‐García, Fernández, Álvarez‐Salgado, et al. 2010; Teira et al. 2010). However, it had been suggested that Red Sea SAR11 bacteria, which represent a substantial fraction of the total community (Ngugi et al. 2012), do not utilise glucose as a primary carbon source (Jimenez‐Infante et al. 2017). Rather, more complex organic molecules such as nucleotides containing also N and P would have been preferred in these oligotrophic waters (Sisma‐Ventura and Rahav 2019), which based on our results were co‐limited by both inorganic nutrients (Mills et al. 2008).

The strong positive responses of the abundance of *Synechococcus*, including the LF and HF groups, either after nitrate and phosphate additions alone (I treatment) or when mixed with glucose (M treatment) in most seasons, match observations from the Atlantic Ocean (Joint et al. 2002; Martínez‐García, Fernández, Calvo‐Díaz, et al. 2010). Although *Synechococcus* are potential mixotrophs with the ability to utilise organic resources through osmotrophy (Flynn et al. 2013; Kang et al. 2004), we observed a negative response to the addition of glucose alone in most seasons, suggesting the dominance of strict photoautotrophs at the surface of our site (Muñoz‐Marín et al. 2024). A similar effect was also observed in a microcosm experiment in the E North Sea (Joint et al. 2002). When comparing their RRs, in contrast to *Synechococcus*, the responses of total heterotrophic bacteria to nutrient addition were weak, but still mostly stimulated by inorganic (spring and fall) or mixed (fall) additions (Figure 2B). Although these results agree with the increasing evidence that heterotrophic bacteria can compete with phytoplankton for inorganic nutrient uptake, especially in oligotrophic regions (Joint et al. 2002; Sipura et al. 2005), the most plausible explanation is that the positive response of heterotrophic bacteria to the addition of inorganic N and P was indeed a consequence of fresh DOM being released by nutrient‐stimulated phytoplankton (Sebastián and Gasol 2013). The apparently contradictory lack of response to the addition of glucose only, previously discussed, can be explained by the fact that dissolved organic nitrogen and phosphorus compounds were likely more abundant in the phytoplankton exudates from the I and M treatments than in the O treatment lacking inorganic nutrients (Caron et al. 2000).

The HNFs abundance peak (on average 1.8‐fold higher in the I and M than in the C treatments) appeared usually 1 day after that of heterotrophic bacteria. Although our daily sampling scheme did not allow for a detailed assessment of the temporal coupling between both groups, they support the classical Lotka‐Volterra predator–prey models frequently observed in aquatic ecosystems (e.g., Tanaka and Taniguchi 1999; Sintes and del Giorgio 2014). The timing of the maximum abundances of the two microbial groups indicates a fast response in grazing pressure after heterotrophic bacteria increased their numbers (Calbet et al. 2001). A similar pattern was also reported in a microcosm experiment in the oligotrophic waters of the central Atlantic Ocean after adding organic matter (Jürgens et al. 2000). Since the response of HNFs is frequently linked to the actual conditions of their prey (Matz and Jürgens 2003), the existence of a positive response of HNFs in the I and M treatments during spring (i.e., RR significantly > 1, Figure 2C) suggests that HNFs were more dependent on their prey during this season, as previously shown (Sabbagh et al. 2020, 2023), suggestive of recurrent changes in community composition. Bouvy et al. (2011) also showed marked bacterial growth when inorganic and organic nutrients were added and HNFs were removed, compared to microcosms with HNFs present, confirming that HNFs bacterivory increased in response to prey conditions in the Mediterranean. On the contrary, the lack of a significant positive response of HNFs to nutrient addition in the remaining experiments might point to a fast response of their consumers (ciliates and other microzooplankton that remained in the sample after the pre‐filtration through 200 μm) to increased HNFs numbers (Gasol and Vaque 1993; Gasol 1994; Segovia et al. 2016). A simpler explanation is that the huge discrepancy in the individual HNFs RR values precluded the finding of significant responses in summer and fall, although they were essentially the same as the spring one (Figure 2C).

Viral dynamics did not exhibit as clear a pattern as protistan grazers, but oscillations between the abundances of lytic viruses and their specific hosts were demonstrated to occur in natural assemblages (Riemann and Middelboe 2002). The higher maximum concentrations of viruses found in the I (1.6‐fold) and M (1.2‐fold) treatments relative to the C could be a consequence of the corresponding higher maximum abundances of autotrophic and heterotrophic bacterioplankton in the same treatments. Experimental nutrient manipulation experiments have found a greater response of viruses after inorganic N and P additions alone (Tsiola et al. 2016) or when combined with organic substrates (Øvreås et al. 2003), concomitant with the response of their hosts. The significant positive responses of viral abundance to nutrient additions did not mirror the corresponding RR values greater than 1 of heterotrophic bacteria, suggesting that viral responses to nutrient addition were seasonally variable and/or that bacterial infections did not take place massively at least at the time‐scale of 6 days (Suttle and Chen 1992; Winter et al. 2004). Moreover, the possible presence of bacterial taxa resistant to viral infection could have played a role in their overall response to nutrient additions (Liu et al. 2015), as virus resistant species have been reported to outgrow in eutrophic environments, where host‐phage encounters are more frequent (Wommack and Colwell 2000). Overall, our results demonstrate that external nitrogen and phosphorus inputs into the coastal Red Sea, particularly common near urban areas, ultimately influence pelagic food webs, but the response of viruses was far from straightforward and less seasonally dependent than that of HNFs (Table 3). Other environmental variables like temperature might alter the way planktonic communities respond to nutrient inputs, as discussed below.

*Synechococcus* was the microbial group that showed the highest number of significant relationships between their RRs and temperature over the four seasons (7 out of 12 in Figure 3, Table 3). However, only the positive ones (57%) would agree with Courboulès et al. (2021), who found stronger responses to raised temperature of phytoplankton compared to heterotrophic bacteria, suggesting a potential competitive advantage of the former under nutrient limitation and warmer conditions. Nevertheless, when we considered a higher temperature range by pooling together the 4 experiments (20°C–36°C), we failed to find an apparent effect of temperature on the RRs of *Synechococcus* in any nutrient treatment (Figure 4A), suggesting that once nutrients were added, temperature had only a minor impact on cyanobacteria RRs. Conversely, the lack of significant responses of heterotrophic bacteria RRs to temperature (Figure 4B) may in turn be related to their stronger limitation by substrate availability or higher mortality rates in low latitude regions (Morán et al. 2017, 2020; Gu et al. 2020). It has been suggested that increasing temperature can alter microbial composition through the selection of thermally better‐adapted taxa. For instance, Lindh et al. (2013) observed differences in bacterial community composition in mesocosm experiments conducted in the Baltic Sea, in which members of Betaproteobacteria dominated the bacterial community when temperature increased 3°C, but were overtaken by Bacteroidetes when the increase was 6°C. Accordingly, it is highly plausible that different taxa responded differently to increasing temperature. Interestingly, a recent temperature‐nutrient interaction model suggested that nutrient depletion will likely exacerbate the impact of warming on bacterial communities (Thomas et al. 2017).

Although the response was not evident in the RRs of autotrophic and heterotrophic bacteria, we found significant, positive relationships of the RRs of HNFs and viruses versus pooled temperature in the I treatment (Figure 4C,D). This suggests a rapid consumption and infection of more bacterial prey available due to enhanced primary production following inorganic nutrients uptake, undetected in their RRs but indeed observed in those of their mortality agents. This observation is in agreement with other reports (Peters 1994; Vázquez‐Domínguez et al. 2012) that demonstrated higher bacterial consumption rates by HNFs under warmer conditions. It remains to be explained, though, the significant negative response of HNFs RRs to temperature in the O treatment, although the original RR values were statistically indistinguishable from 1 (i.e., no real effect of glucose addition, Figure 2C). Regardless of the actual nutrient or temperature treatments, our study confirms a tight coupling of HNFs and their bacterial prey in the Red Sea through their RRs along the whole year except winter (Figure 5A). Despite the lack of clear, direct relationships between the RRs of the various microbial groups and temperature (Figure 4), warmer temperatures did strengthen the interaction between heterotrophic bacterioplankton and their protistan grazers (Figure 5B). The significant correlation between the temperature dependence of their maximum abundances shown in Table 2 (i.e., higher values of maximum heterotrophic bacteria abundance change with temperature were accompanied by higher values of maximum HNFs cells °C^−1^) points also in the same direction. Indeed, 44% of the variance in the percent change per °C increase in the maximum abundance of HNFs could be solely explained by the equivalent of heterotrophic bacteria (HB) (%HNF_max_ °C^−1^ = 0.15 + 3.17%, HB_max_ °C^−1^, *r* ^2^ = 0.44, *p* = 0.005, *n* = 16). Although we did not measure grazing rates of bacteria by HNFs in this study, other experimental work showed higher values in warmer conditions (Peters 1994; Bouvy et al. 2011; Vázquez‐Domínguez et al. 2012), consistent with the response observed in our study.

The fact that the RRs of viruses were positively correlated to experimental temperature with data pooled from the I and M treatments (Figure 4D) could be interpreted within the killing‐the‐winner hypothesis (Thingstad 2000). These results also align with the suggestion that increasing temperature will likely influence the virus‐host interactions (Danovaro et al. 2011) by increasing their contact rates, potentially leading to higher host lysis (Murray and Jackson 1992; Bettarel et al. 2009). Interestingly, the positive relationship between the RR of viruses and the RR of bacteria in summer (Figure 5C) coincides with the period of the year in which viral impact was maximum at KAUST Harbor (Sabbagh et al. 2020, 2023). However, the strength of the relationship of the coupling between the RRs of viruses and bacteria with in situ temperature (Figure 5D) was not evident, although only the winter value prevented it from being significant as in the case of HNFs (Figure 5B). Future responses of microbial interactions to environmental stressors are complex to estimate only from this type of experimental work, but our results suggest that ongoing warming in the Red Sea might result in tightening of the trophic dependence of protistan grazers (and to a lower extent viruses) on their heterotrophic bacterial prey.

## 5 Conclusion

While this study provides valuable insights into the potential effect of nutrient addition and warming on the bottom‐up and top‐down controls of Red Sea bacterioplankton assemblages, limitations are acknowledged. We inferred their impact based on measurements of microbial abundances and the calculation of RRs rather than on direct growth and mortality rates. In addition, correlations suggest potential relationships between microbial abundances and changes in nutrients and temperature but do not necessarily establish causation. Future studies should incorporate direct measurements of microbial activities to better understand the dynamics of microbial food webs under the pressure of environmental stressors in the Red Sea. Notwithstanding the mentioned limitation, the manipulation of nutrient concentrations and temperatures in 4 experiments covering a full seasonal cycle in shallow waters of the central Red Sea revealed a clear effect of inorganic and mixed nutrient additions through increased abundances of photosynthetic microbes, while the response to organic nutrient (glucose) additions was either minor or negative. Based on this finding, efforts at precluding eutrophication of Red Sea coastal waters should be maintained. Short‐term experimental warming exerted weak and inconsistent responses on microbial communities within each seasonal assessment, but the temperature dependence of bacterioplankton top‐down control was more pronounced when considering the whole range of the study (20°C–36°C). The existence of positive relationships between the nutrient‐amendment responses of bacteria and HNFs abundance during most seasons and between bacteria and viruses in summer would confirm previous studies suggesting the key role of top‐down control in keeping bacterioplankton stocks low at the Red Sea, even when macronutrients were added. Although inorganic nutrient inputs had a greater impact than warming on microbial abundances, the potential coupling between the responses of heterotrophic bacteria and HNFs became tighter at higher in situ temperatures. Given the currently hot temperatures of the coastal central Red Sea (exceeding 34°C in summer), the enhancement of the bacteria‐HNFs trophic linkage with further warming might result in a strengthening of the microbial loop (Azam et al. 1983) at the expense of the larger phytoplankton pathways.

## Author Contributions

**Eman I. Sabbagh:** conceptualization, data curation, formal analysis, visualization, writing – original draft, methodology, investigation, writing – review and editing, validation. **Najwa Al‐Otaibi:** conceptualization, data curation, formal analysis, methodology, writing – review and editing. **Maria Ll. Calleja:** conceptualization, data curation, formal analysis, writing – review and editing, methodology, investigation. **Daniele Daffonchio:** writing – review and editing, funding acquisition. **Xosé Anxelu G. Morán:** conceptualization, data curation, formal analysis, visualization, methodology, investigation, supervision, writing – review and editing, funding acquisition, resources.

## Disclosure

We confirm that the work presented in this manuscript is original and complies with all applicable local, national and international regulations, conventions and standard scientific ethical practices.

## Conflicts of Interest

The authors declare no conflicts of interest.

## Supporting information


**Figure S1:** Dynamics of the contribution (%) of low fluorescence *Synechococcus* (LF *Synechococcus*, A–D), high nucleic acid bacteria (HNA, E–H) and low nucleic acid viruses (V1, I–L) at in situ temperature in the different nutrient treatments. C, control; I, inorganic; M, mixed; O, organic. Error bars represent SD of duplicate samples.

**Figure S2:** Mean seasonal contribution (%) of the response ratio (RR) of (A) low fluorescence *Synechococcus* (LF *Synechococcus*), (B) high fluorescence *Synechococcus* (HF *Synechococcus*), (C) high nucelic acid bacteria (HNA), (D) low nucleic acid bacteria (LNA), (E) low nucleic acid viruses (V1), (F) medium nucleci acid viruses (V2) and (G) high nucleic acid viruses (V3) at in situ temperature in the different nutrient addition treatments (codes as in Figure S1). Error bars represent the SD of duplicate samples. The horizontal line represents a RR of 1.0 (no change relative to the C treatment). Asterisks indicate a RR significantly different from 1 (*t*‐test: *p < 0.05; **p < 0.01; ***p < 0.001).

## Data Availability

The data that support the findings of this study are available on request from the corresponding author. The data are not publicly available due to privacy or ethical restrictions.
