# Supplementary figures and images for: Changes in the Top‐Down Control of Planktonic Bacteria in Response to Nutrient Addition and Warming in the Red Sea
Source: Environ Microbiol Rep. 2025 Aug 2;17(4):e70166. doi: 10.1111/1758-2229.70166 (PMC12317192; doi:10.1111/1758-2229.70166)

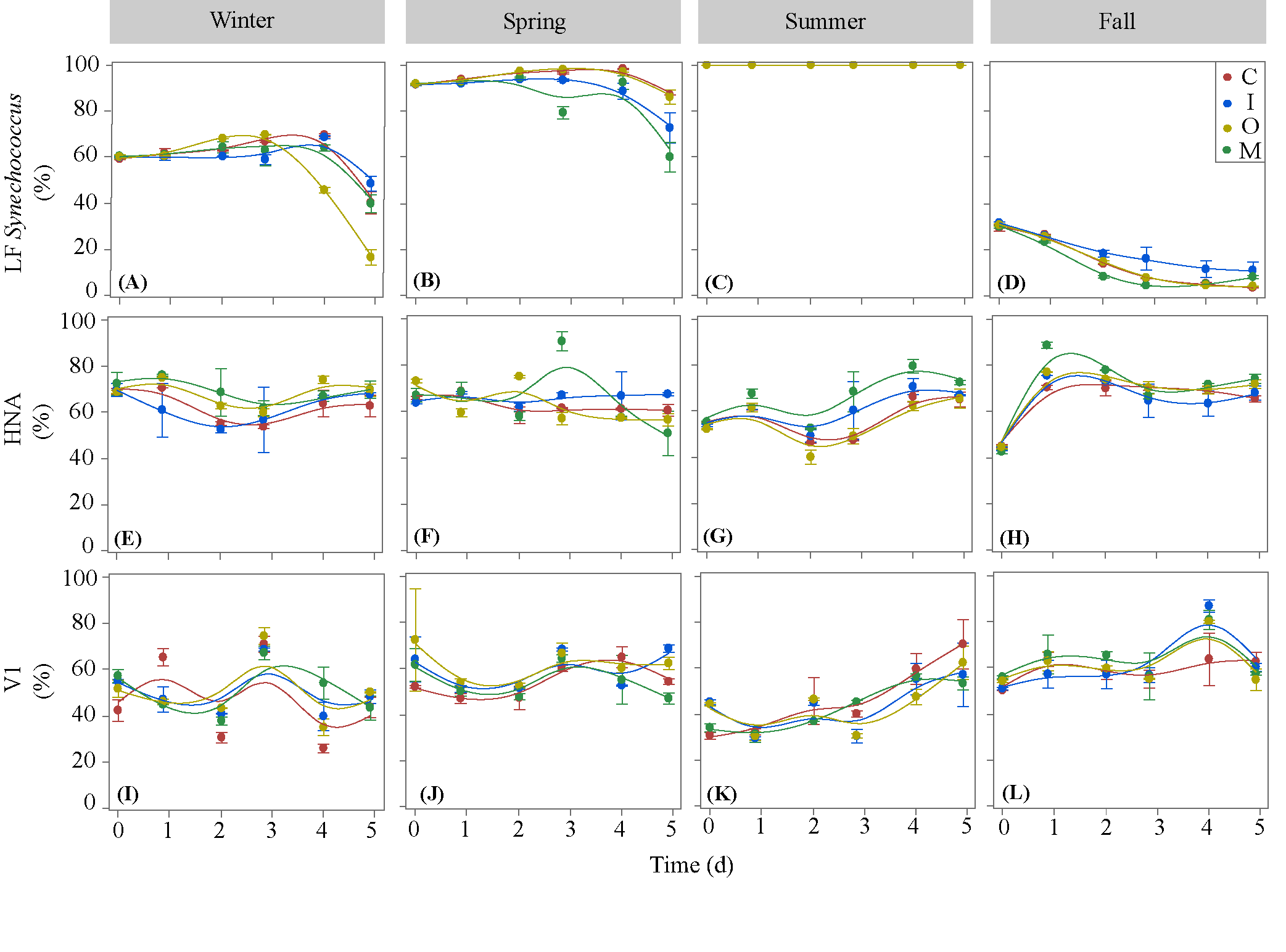

Supplement: Supplementary file 1 — Figure S1: Dynamics of the contribution (%) of low fluorescence Synechococcus (LF Synechococcus, A–D), high nucleic acid bacteria (HNA, E–H) and low nucleic acid viruses (V1, I–L) at in situ temperature in the different nutrient treatments. C, control; I, inorganic; M, mixed; O, organic. Error bars represent SD of duplicate samples. [file EMI4-17-e70166-s002.tif]

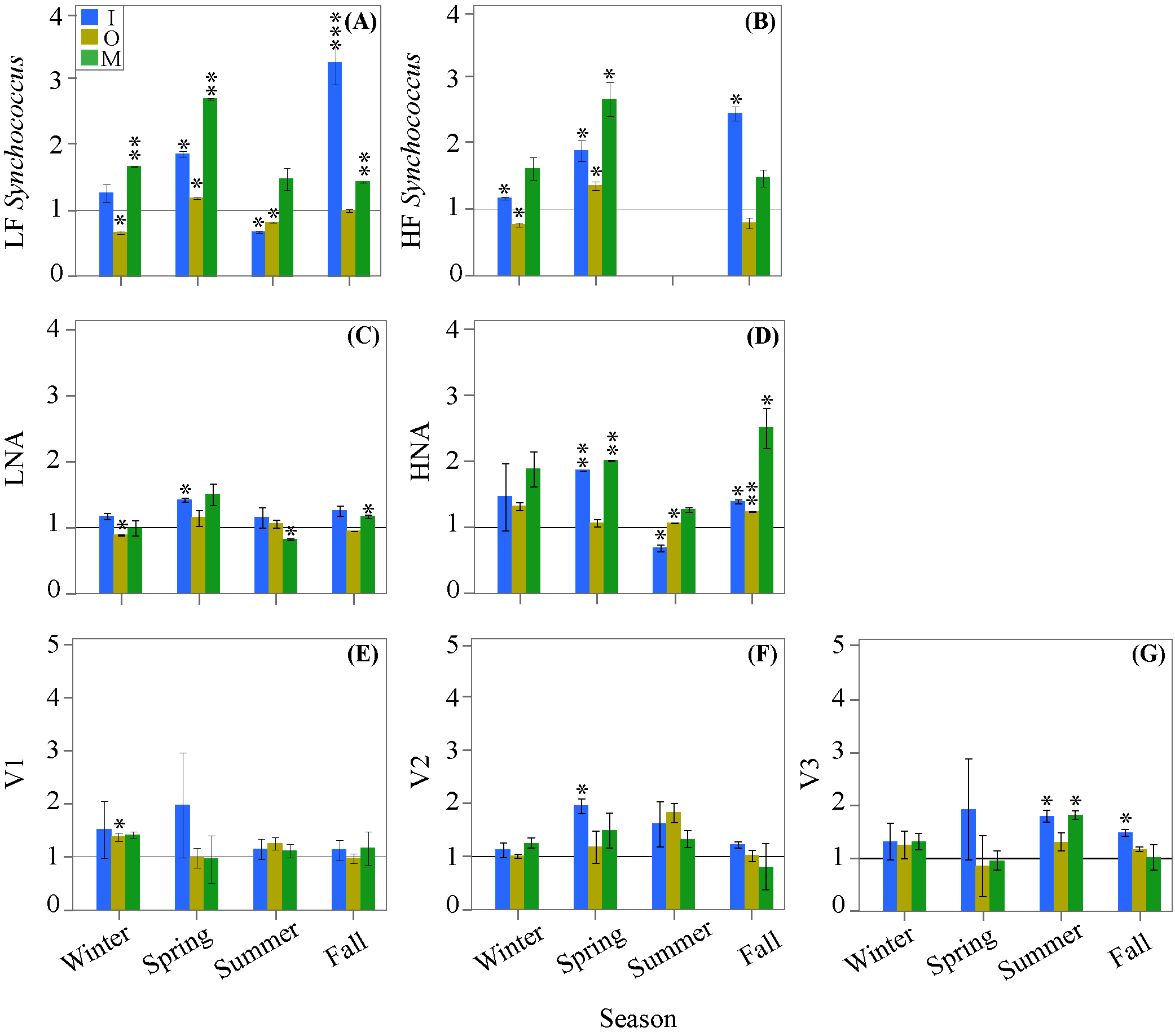

Supplement: Supplementary file 2 — Figure S2: Mean seasonal contribution (%) of the response ratio (RR) of (A) low fluorescence Synechococcus (LF Synechococcus), (B) high fluorescence Synechococcus (HF Synechococcus), (C) high nucelic acid bacteria (HNA), (D) low nucleic acid bacteria (LNA), (E) low nucleic acid viruses (V1), (F) medium nucleci acid viruses (V2) and (G) high nucleic acid viruses (V3) at in situ temperature in the different nutrient addition treatments (codes as in Figure S1). Error bars represent the SD of duplicate samples. The horizontal line represents a RR of 1.0 (no change relative to the C treatment). Asterisks indicate a RR significantly different from 1 (t‐test: *p < 0.05; **p < 0.01; ***p < 0.001). [file EMI4-17-e70166-s001.tif]
